# Development of a Comparative European Orthohantavirus Microneutralization Assay With Multi- Species Validation and Evaluation in a Human Diagnostic Cohort

**DOI:** 10.3389/fcimb.2020.580478

**Published:** 2020-12-22

**Authors:** Tabitha E. Hoornweg, Ilse Zutt, Ankje de Vries, Miriam Maas, Marieke N. Hoogerwerf, Tatjana Avšič-Županc, Miša Korva, Johan H. J. Reimerink, Chantal B. E. M. Reusken

**Affiliations:** ^1^ Centre for Infectious Disease Control, National Institute for Public Health and the Environment (RIVM), Bilthoven, Netherlands; ^2^ Institute of Microbiology and Immunology, Faculty of Medicine, University of Ljubljana, Ljubljana, Slovenia

**Keywords:** orthohantaviruses, Dobrava virus, Puumala virus, Seoul virus, Tula virus, virus neutralization test, microneutralization test, serotyping

## Abstract

Orthohantaviruses (family *Hantaviridae*, order *Bunyavirales*) can cause two serious syndromes in humans: hemorrhagic fever with renal syndrome (HFRS), associated with the Old World orthohantaviruses, and hantavirus cardiopulmonary syndrome (HCPS), associated with orthohantaviruses in the Americas. In Europe, four different orthohantaviruses (DOBV, PUUV, SEOV, and TULV) are associated with human disease. As disease severity and zoonotic source differ between orthohantavirus species, conclusive determination of the infecting species by either RT-PCR or comparative virus neutralization test (VNT) is of importance. Currently, the focus reduction neutralization test (FRNT) is considered the ‘Gold Standard’ for orthohantavirus VNTs, however this test is laborious and time-consuming. Consequently, more high-throughput alternatives are needed. In this study, we developed a comparative orthohantavirus microneutralization test (MNT) including all four human pathogenic orthohantavirus species circulating in Europe. The assay was validated using RT-PCR-confirmed rodent (n=17) and human sera (n=17), DOBV-suspected human sera (n=3) and cohorts of orthohantavirus-negative rodent (n=3) and human sera (n=85). 16/17 RT-PCR-confirmed rodent sera and 18/20 of the RT-PCR-confirmed and DOBV-suspected human sera were serotyped successfully, while for the remaining rodent (n=1) and human sera (n=2) no neutralizing titers could be detected. All negative control sera tested negative in the MNT. The assay was subsequently evaluated using a clinical cohort of 50 orthohantavirus patients. Orthohantavirus infection was confirmed in all 50 patients, and 47/50 (94%) sera were serotyped successfully, confirming PUUV as the major cause of orthohantavirus infections in Netherlands. Notably, two previously unrecognized SEOV cases from 2013 were diagnosed using the MNT, underlining the added value of the MNT in a diagnostic setting. In conclusion, we demonstrate the successful development and clinical implementation of a comparative European orthohantavirus MNT to determine the infecting virus species in European HFRS patients. Identification of the causative species is needed for an adequate Public Health response and can support individual patient care. For many labs, the implementation of orthohantavirus neutralization tests has not been a straightforward procedure. This issue will be addressed by the rollout of the comparative MNT to multiple European laboratories to support patient diagnostics, surveillance and Public Health responses.

## Introduction

Orthohantaviruses are enveloped negative stranded RNA viruses with a tripartite genome that belong to the family of *Hantaviridae*, order *Bunyavirales* (Reuter and Kruger, 2018). Each orthohantavirus species is carried by one or few closely related species of reservoir host(s), either rodents, shrews, moles or bats (Vapalahti et al., 2003; Reusken and Heyman, 2013). As a consequence the distribution of orthohantaviruses is strictly dependent on the dispersal of their reservoir host. Although orthohantaviruses typically cause chronic infections without overt disease in their reservoir host (Vaheri et al., 2013), in humans they may cause serious illness. While old world orthohantaviruses are mainly associated with hemorrhagic fever with renal syndrome (HFRS), new world orthohantaviruses are primarily associated with hantavirus cardiopulmonary syndrome (HCPS). However, it is increasingly being recognized that considerable clinical overlap exists between both syndromes (Clement et al., 2014).

Four different orthohantavirus species are associated with human disease in Europe: Dobrava/Belgrade virus (DOBV) carried by three closely related species of the genus *Apodemus;* Puumala virus (PUUV) carried by the bank vole (*Myodes glareolus*); Seoul virus (SEOV) carried by different species of rats (*Rattus* spp.); and Tula virus (TULV) carried by the common vole (*Microtus arvalis*) (Reusken and Heyman, 2013). Additionally, while Hantaan virus (HTNV), carried by the striped field mouse *(A. agrarius)*, is generally believed to be only present in Asia, some studies suggested that it may also be present in eastern parts of Europe (Clement et al., 1997).

Of the orthohantaviruses with confirmed presence in Europe, DOBV is associated with the most severe form of HFRS with case fatality ratio’s (CFR) up to 12%, depending on the genotype (Klempa et al., 2013). SEOV is associated with a mild-moderate form of HFRS, whereas PUUV causes a relatively mild form of HFRS, also known as Nephropathia Epidemica (NE) (Vapalahti et al., 2003; Kruger et al., 2011; Reusken and Heyman, 2013). To date, TULV has only been associated with a low number of human infections, which were either considered asymptomatic or in which a mild form of HFRS was observed (Vapalahti et al., 1996; Schultze et al., 2002; Clement et al., 2003; Klempa et al., 2003; Zelena et al., 2013; Reynes et al., 2015).

In Netherlands, PUUV, SEOV and TULV all circulate in their respective reservoir hosts (Reusken et al., 2008; Verner-Carlsson et al., 2015; de Vries et al., 2016; Maas et al., 2017; Swanink et al., 2018). Each year approximately 25–30 human orthohantavirus infections are reported, although this is likely an underestimation (Goeijenbier et al., 2014). Due to the high genetic variability within the orthohantavirus species and the notion that in many cases no viral RNA can be detected in the first clinical samples sent to diagnostic laboratories, laboratory diagnostics are primarily based on serology (Vapalahti et al., 2003). The pathogenic orthohantaviruses present in Europe can be subdivided into two different serogroups (Vaheri et al., 2008); PUUV and TULV belong to the serogroup of *Arvicolinae*-borne orthohantaviruses, while DOBV and SEOV (together with HNTV) are part of the serogroup of *Murinae*-borne orthohantaviruses. Unfortunately, however, as high levels of cross-reactive antibodies are observed within but also between the different serogroups (albeit to a lesser extent), the infecting virus species cannot be determined conclusively using routine serological assays (Vapalahti et al., 2003). As a consequence, in Netherlands, orthohantavirus infections are routinely attributed to PUUV which is assumed to be the most widespread orthohantavirus in the country (Sane et al., 2014). Yet, as disease severity and the zoonotic source differ between orthohantavirus species, conclusive determination of the infecting species is of importance for surveillance, source attribution, risk management and monitoring of control measures.

Species determination of the infecting orthohantavirus can only be done by RT-PCR or comparative virus neutralization assays (VNT). To date, multiple variations of orthohantavirus neutralization assays have been described (Tanishita et al., 1984; Lee et al., 1985; Takenaka et al., 1985; Niklasson et al., 1991; Horling et al., 1992; Chu et al., 1995; Heider et al., 2001; Maes et al., 2009; Padua et al., 2015; Li et al., 2017), however, the focus reduction neutralization test (FRNT) is generally viewed as the ‘Gold Standard’. Unfortunately, the orthohantavirus FRNT is rather laborious and time consuming and therefore not very suitable for assaying a large number of samples (Horling et al., 1992; Li et al., 2017). Recently, Li et al. (2017) showed that the more high-throughput microneutralization test (MNT) was a good alternative to the FRNT when serotyping human infections with the closely related HTNV and SEOV (Li et al., 2017). In this study, we describe the development, validation and evaluation of a comparative orthohantavirus MNT including all four human pathogenic orthohantavirus species circulating in Europe.

## Materials and Methods

### Cells, Viruses, and Antibodies

Green monkey kidney Vero-E6 cells (ATCC C1008 CRL1586) were maintained in Dulbecco’s Modified Eagle’s MEM (DMEM, Gibco) supplemented with 5% heat-inactivated fetal bovine serum (FBS; Biowest, Nuaillé, France), 100 U/ml penicillin and 100 U/ml streptomycin (Biowest, Nuaillé, France). Cells were cultured at 37°C under 5% CO2.

DOBV strain SK/Aa (catalog number: 008v-EVA1470), PUUV strain Sotkamo (catalog number: 008v-EVA1472), SEOV strain 80–39 (catalog number: 008v-EVA1473), and TULV strain Moravia (no longer available from EVAg) were all acquired *via* the European Virus Archive (EVAg). Stocks were created by passaging twice on Vero-E6 cells using a low multiplicities of infection (MOI 0.001-0.1), and harvesting 7–14 days post infection.

### Virus Titration

Titers of the virus stocks were determined by use of a 50% tissue culture infectious dose (TCID_50_) assay adapted for indirect detection of virus-infected cells. Briefly, tenfold viral dilutions were inoculated on Vero-E6 cells and incubated for 7 (DOBV, SEOV and TULV) or 12 days (PUUV) at 37°C, which corresponded to peak titers during virus culture (data not shown). Subsequently, cells were fixed using 4% PFA and permeabilized using 0.02% Triton X-100 in PBS. Subsequently, cells were washed with PBS and stained using mouse anti-hanta nucleoprotein (1:500; abcam, Cambridge, United Kingdom) (for PUUV, SEOV and TULV) or mouse anti-hantavirus Dobrava strain nucleoprotein (1:1,000; IBTsystems, Binzwangen, Germany) (for DOBV) as primary antibodies and goat anti-mouse-AlexaFluor488 (1:200; Abcam, Cambridge, United Kingdom) as a secondary antibody. Positive wells were visualized using a Leica DMIL LED fluorescence microscope and scored by eye. TCID_50_ titers were calculated using the Spearman and Kärber algorithm (Hierholzer and Killington, 1996).

### Microneutralization Test

The orthohantavirus neutralization test was designed as a microneutralization tests (MNT) as previously described for the Influenza A virus and the orthohantaviruses HTNV and SEOV (Klimov et al., 2012; Li et al., 2017) with an adaptation for a fluorescence-based read-out. Briefly, two-fold serial dilutions (starting dilution 1:40) of heat-inactivated sera (30 min at 56°C) were mixed with 100 TCID_50_ of the appropriate virus species and incubated for 2 h at 37°C. Subsequently, 100 µl virus-antibody complexes were transferred to a 96-wells plate containing a 90% confluent Vero-E6 monolayer. 1.5–3 h post infection, an additional 100 µl Vero-E6 medium supplemented with 25 mM HEPES was added to each well. Plates were incubated for 7 (DOBV, SEOV, TULV) or 12 (PUUV) days at 37°C, before cells were fixed, permeabilized and stained as described above. Virus-positive cells were visualized using a Leica DMIL LED fluorescence microscope. The neutralization titer was determined by scoring the wells in which infection was inhibited ≥ 95% compared to the positive control (MNT_95_).

### Serum Panels for Validation of the Orthohantavirus MNT

For validation of the orthohantavirus MNT, a panel of 17 rodent sera representing the four different human pathogenic European orthohantavirus species and their associated reservoir was used, of which all specifications are included in **Table 1**. The rodent sera were both RT-PCR and IgG positive using routine serology for DOBV (n=4), PUUV (n=5), SEOV (n=5), or TULV (n=3). The sera positive for DOBV, PUUV, and TULV originated from wild rodents caught for surveillance purposes in either Netherlands or Slovenia. The SEOV positive sera came from a SEOV-positive feeder rat colony associated with a human SEOV infection in Netherlands (Swanink et al., 2018)). Additionally, a small panel of wild caught *R. norvegicus* sera (n=3) that previously tested negative in both RT-PCR and the orthohantavirus FRNT in Uppsala, Sweden (Maas et al., 2018) was included as negative controls. All animal handling procedures were approved by the Dutch (DEC project numbers 200900164 and 201200208) and Slovenian Animal Ethics Committee (Administration for Food Safety, Veterinary Sector and Plant Protection: U34401-15/2018/8).

**Table 1:** Specifics of the rodent serum panel used for validation of the orthohantavirus microneutralization test (MNT).

Serum #	Host	Country of Origin	Serology			RT-(q)PCR		
			+/–	Test	Value	+/−	Segment	Species
PUUV 1	*Myodes Glareolus*	Netherlands	+	PUUV-IgG immunochromatography fast test	185	+	partial L	PUUV
PUUV 2	*Myodes Glareolus*	Netherlands	+	PUUV-IgG immunochromatography fast test	168	+	partial L	PUUV
PUUV 4	*Myodes Glareolus*	Netherlands	+	PUUV-IgG immunochromatography fast test	129	+	partial L	PUUV
PUUV 6	*Myodes Glareolus*	Slovenia	+	IFT PUUV/IFT DOBV	+++/+++	+	partial S + partial L	PUUV
PUUV 7	*Myodes Glareolus*	Slovenia	+	IFT PUUV/IFT DOBV	++/+/−	+	partial S + partial L	PUUV
TULV 1	*Microtus Arvalis*	Netherlands	+	PUUV-IgG immunochromatography fast test	12	+	partial S	TULV
TULV 2	*Microtus Arvalis*	Netherlands	+	PUUV-IgG immunochromatography fast test	13	+	partial S	TULV
TULV 3	*Microtus Arvalis*	Netherlands	+	PUUV-IgG immunochromatography fast test	10	+	partial S	TULV
rDOBV 1	*Apodemus flavicollis*	Slovenia	+	IFT PUUV/IFT DOBV	−/+	+	partial S + partial L	DOBV
rDOBV 2	*Apodemus flavicollis*	Slovenia	+	IFT PUUV/IFT DOBV	+/++	+	partial S + partial L	DOBV
rDOBV 3	*Apodemus flavicollis*	Slovenia	+	IFT PUUV/IFT DOBV	++/+++	+	partial S + partial L	DOBV
rDOBV 4	*Apodemus agrarius*	Slovenia	+	IFT PUUV/IFT DOBV	+/+	+	partial S + partial L	DOBV-Kurkino
SEOV 1	*Rattus Norvegicus*	Netherlands	+	adapted Hantavirus Dobrava/Hantaan IgG Elisa	2.567	+	partial S	HTNV/SEOV
SEOV 2	*Rattus Norvegicus*	Netherlands	+	adapted Hantavirus Dobrava/Hantaan IgG Elisa	2.48	+	partial S	HTNV/SEOV
SEOV 3	*Rattus Norvegicus*	Netherlands	+	adapted Hantavirus Dobrava/Hantaan IgG Elisa	2.385	+	partial S	HTNV/SEOV
SEOV 4	*Rattus Norvegicus*	Netherlands	+	adapted Hantavirus Dobrava/Hantaan IgG Elisa	2.276	+	partial S	HTNV/SEOV
SEOV 5	*Rattus Norvegicus*	Netherlands	+	adapted Hantavirus Dobrava/Hantaan IgG Elisa	2.121	+	partial S	HTNV/SEOV

The Puumala virus (PUUV)-IgG immunochromatography fast test, the PUUV and different orthohantaviruses (DOBV) IFT, and the adapted Hantavirus Dobrava/Hantaan IgG ELISA have previously been described in (de Vries et al., 2016), (Korva et al., 2013a) and (Swanink et al., 2018), respectively. Reported values either represent the quantified antibody levels as measured by the ReaScan Reader (PUUV-IgG immunochromatography fast test) or OD (adapted Hantavirus Dobrava/Hantaan IgG ELISA). For the IFT a semi-quantitative read-out was used, in which samples were scored −, +/−, +, ++, or +++ based on staining pattern and brightness. RT-(q)PCR protocols were previously described by (Razzauti et al., 2009) (PUUV partial L), (Korva et al., 2013a) (PUUV and DOBV partial S and partial L), and (Kramski et al., 2007) (TULV and HTNV/SEOV partial S). A selection of samples diagnosed by the PUUV partial L and the HTNV/SEOV partial S RT-qPCR protocols were further confirmed by Sanger sequencing.

For the validation of the MNT with human sera, a serum panel from 17 patients with a RT-qPCR confirmed orthohantavirus infection diagnosed in the period of 2011-2017, was used. Eleven sera (9x PUUV-positive, 2x SEOV-positive) originated from Dutch patients and were diagnosed using the genotype-specific RT-qPCRs described by (Kramski et al., 2007), while six sera (5x DOBV-positive, 1x PUUV-positive (Korva et al., 2013b)) originated from Slovenian patients and were diagnosed with RT-qPCRs previously described by (Korva et al., 2009). For 11 patients the acute phase RT-qPCR-positive sample was directly tested in the MNT, while for six patients (3× DOBV-positive, 2× PUUV-positive, 1× SEOV-positive) a subsequent paired serum sample to the RT-qPCR-positive serum sample was tested in the MNT (paired sera are indicated by a * in **Table 4**). Of the paired sera, all three sera from the DOBV-positive patients were late convalescent, while the sera from the PUUV- (2×) and SEOV-positive (1×) patients were drawn less than one month after the onset of symptoms. All patients consented that the remainder of their sera could be used for research purposes.

In addition, a small serum panel from DOBV-suspected human patients (n=3) kindly provided by Sabine Lederer from Euroimmun (Lubeck, Germany) was tested in the orthohantavirus MNT. All samples showed the highest IgG titers against DOBV in the Euroimmun mosaic immunofluorescent assay (IFA) (Lederer et al., 2013), with a varying degree of cross-reactivity toward the other (primarily *Murinae*-borne) orthohantaviruses. Two of three sera were known to be convalescent (both samples were taken more than one year after onset of symptoms), while for the third serum the status was unknown. None of these sera were confirmed by RT-PCR.

Finally, 85 randomly selected sera collected from healthy Dutch blood donors in 2016 were included as negative controls.

### Diagnostic Cohort

Sera of 50 Dutch patients clinically suspected of an orthohantavirus infection, collected in the period 2013–2017, and diagnosed with an orthohantavirus infection based on routine serology were selected for testing in the orthohantavirus MNT. Initial routine orthohantavirus diagnostics were performed using the Euroimmun mosaic IFA (Lederer et al., 2013), in which samples are simultaneously tested against six clinically important orthohantaviruses (DOBV, HTNV, PUUV, Saaremaa virus (SAAV), SEOV and Sin Nombre Virus (SNV)), according to manufacturer’s instructions (Euroimmun, Lubeck, Germany). Sera were considered positive for orthohantavirus infection when IgM and/or IgG titers were ≥128 for at least one of the orthohantaviruses tested (Lederer et al., 2013; Geurts van Kessel et al., 2016). All serum samples were tested negative for the presence of DOBV, PUUV, SEOV/HTNV, and TULV RNA using the serotype-specific RT-qPCRs described by (Kramski et al., 2007). Further specifications of the cohort are listed in **Table 2**. For all samples, the orthohantavirus MNT was performed in parallel for all four orthohantaviruses species. When paired sera were available, the serum sample of the latest collection date was tested in the MNT. All patients consented that the remainder of their sera could be used for research purposes.

**Table 2:** General characteristics of the diagnostic cohort of clinically suspected human orthohantavirus cases diagnosed with anorthohantavrus infection based on routine serology.

Variable	Orthohantavirus infections
	(n=50)
Gender - No. (%)	
Male	35 (70.0)
Female	15 (30.0)
Age - years	
Median	46
Range	15–77
Age group - No. (%)	
< 20 years	4 (8.0)
21–40 years	17 (34.0)
41–60 years	19 (38.0)
> 61 years	10 (20.0)
Serogroup with highest reactivity in IFA - No. (%)	
PUUV/SNV	37 (74.0)
DOBV/HTNV/SAAV/SEOV	4 (8.0)
No difference*	9 (18.0)
Serum collection (days post onset of infection) - No. (%)	
<30	16 (32.0)
>30	6 (12.0)
Unknown	28 (56.0)
Travel History - No. (%)	
Yes	5 (10.0)
No	1 (2.0)
Unknown	44 (88.0)

A * indicates that at the highest dilution tested, samples were IgM and IgG positive for both serogroups.

## Results

### Development of a Fluorescent-Based “European” Orthohantavirus Microneutralization Assay

The aim of this work was to set up a MNT including all human pathogenic orthohantaviruses circulating in Europe, namely DOBV, PUUV, SEOV, and TULV. Initially, we designed the European orthohantavirus MNT according to the approach of (Li et al., 2017), in which infection is quantified by measuring the enzymatic activity of horseradish peroxidase (HRP). However, as for both PUUV and TULV only relatively low OD values could be measured under positive control conditions (infection with 100 TCID_50_), it was not possible to determine levels of neutralization of PUUV and TULV infection robustly using this approach (data not shown).

Consequently, the MNT was designed with a fluorescence-based read-out, in which the MNT_95_ titer, corresponding to the highest antibody dilution that inhibited infection ≥ 95%, was determined by eye. As shown in **Supplementary Figure 1**, virus-infected cells could be clearly discerned for all orthohantaviruses used in this study. The MNT_95_ titer was preferred over an end-point neutralization titer (complete inhibition of infection), as at times small breakthrough infections could be observed that prevented the determination of an end-point neutralization titer, even though clear neutralization was observed.

### Validation: RT-PCR-Confirmed Rodent Sera

For initial validation of the MNT assay, a panel of sera (n=17) from multiple rodent species, previously determined to be serologically (IgG) and RT-PCR positive for either DOBV, PUUV, SEOV or TULV, was used. **Table 3** shows the MNT_95_ titers of the rodent serum validation panel. Neutralization titers of ≥ 40 were considered positive (Kallio-Kokko et al., 2006; Hofmann et al., 2014). Additionally, when positive MNT_95_-titers against multiple orthohantavirus species were found, a titer difference of ≥4 in the comparative tests was required for definitive serotyping (Nichol et al., 2005).

**Table 3:** MNT_95_ titers of rodent sera used for orthohantavirus microneutralization test (MNT) validation.

Serum #	Host	Orthohantavirus MNT_95_					
		95% neutralization titer				Minimal Fold change	Conclusion
		PUUV	TULV	DOBV	SEOV		
PUUV 1	*Myodes glareolus*	≥ 5,120	160	< 40	<40	≥ 32	PUUV
PUUV 2	*Myodes glareolus*	≥ 5,120	160	< 40	< 40	≥ 32	PUUV
PUUV 4	*Myodes glareolus*	≥ 5,120	80	< 40	< 40	≥ 64	PUUV
PUUV 6	*Myodes glareolus*	≥ 5,120	< 40	<40	< 40	> 128	PUUV
PUUV 7	*Myodes glareolus*	≥ 5,120	< 40	< 40	< 40	> 128	PUUV
TULV 1	*Microtus arvalis*	80	2,560	< 40	< 40	32	TULV
TULV 2	*Microtus arvalis*	80	2,560	< 40	< 40	32	TULV
TULV 3	*Microtus arvalis*	640	≥ 5,120	< 40	< 40	≥ 8	TULV
rDOBV1	*Apodemus flavicollis*	< 40	< 40	320	< 40	≥ 16	DOBV
rDOBV2	*Apodemus flavicollis*	< 40	< 40	80	< 40	≥ 4	DOBV
rDOBV3	*Apodemus flavicollis*	< 40	< 40	640	< 40	≥ 32	DOBV
rDOBV4	*Apodemus agrarius*	< 40	< 40	< 40	< 40	–	Negative
SEOV 1	*Rattus norvegicus*	80	< 40	1,280	≥ 5,120	≥ 4	SEOV
SEOV 2	*Rattus norvegicus*	40	< 40	< 40	≥ 5,120	≥ 128	SEOV
SEOV 3	*Rattus norvegicus*	80	< 40	320	1,280	4	SEOV
SEOV 4	*Rattus norvegicus*	80	< 40	320	≥ 5,120	≥ 16	SEOV
SEOV 5	*Rattus norvegicus*	< 40	< 40	640	≥ 5,120	≥ 8	SEOV

Neutralization titers of ≥ 40 were considered positive and, when neutralizing titers against multiple orthohantaviruses were detected, a titer difference of ≥ 4-fold was required for definitive serotyping. The highest positive titer is highlighted in gray.

Close to all rodent sera (16/17) had distinct neutralizing titers toward the infecting virus species and could be serotyped as expected (**Table 3**). For one of the rodent sera positive for DOBV (rDOBV4, derived from *A. agrarius*), no neutralization was observed. As this serum was only weakly positive in the IFT, it is possible that the serum was taken early after infection of the respective rodent and neutralizing antibodies were not yet present at the time of sampling.

For most sera (n=11/17), some degree of cross-neutralization toward the non-infecting other orthohantaviruses was observed, which was most prominent toward the orthohantaviruses from the same serogroup (PUUV ↔ TULV and SEOV → DOBV) (Vaheri et al., 2008). Cross-neutralization toward viruses belonging to the other serogroup (PUUV/TULV ↔ DOBV/SEOV) was generally low (maximal cross-reactive titer observed: 1280 for SEOV, 80 for PUUV (SEOV3)), and only observed for the SEOV-positive sera. Nevertheless, a ≥ 4-fold difference in neutralizing titers was observed for all MNT positive sera, and the infecting virus could be confidently serotyped for each serum.

As a negative control, wild-caught *R. norvegicus* sera that were found to be positive in a genus-wide orthohantavirus ELISA but were previously determined to be negative in both RT-PCR and an orthohantavirus FRNT (Maas et al., 2018), were tested in the MNT. In line with the FRNT results, all sera were negative in the MNT_95_ (data not shown).

### Validation: RT-PCR-Confirmed Human Patient Sera

Next, we aimed to validate the orthohantavirus MNT using sera of RT-qPCR-confirmed human orthohantavirus cases (n=17; 11 acute sera and six paired serum samples of a later sampling date). For this purpose, we used sera from 11 Dutch and six Slovenian patients with a RT-qPCR-confirmed orthohantavirus infection diagnosed in the period from 2011 to 2017. Of these patients, 10 were RT-qPCR positive for PUUV (9 Dutch patients and 1 Slovenian patient), two were positive for SEOV (both Dutch patients) and five were positive for DOBV (all Slovenian patients). As human TULV infections are rare, no sera from RT-qPCR confirmed TULV patients were available.

Neutralizing titers for the RT-qPCR-confirmed patient sera are shown in **Table 4**. All PUUV (n=10) and DOBV (n=5) cases could be serotyped correctly using the orthohantavirus MNT. For SEOV, however, both cases were negative in the MNT. One of these sera (17-07b) did show visible neutralization at the lowest antibody dilutions (i.e. 40 and 80); however the extent of neutralization did not reach 95%. Although this serum sample was a paired sample to a previous RT-qPCR-positive serum sample and showed clear IgM and IgG titers in the diagnostic IFA, the exact period between onset of symptoms and serum sampling were unknown. Consequently, it remains unknown whether the sample tested was either acute or (early) convalescent. For the other serum (17-08), no neutralization was observed. This serum was an acute phase serum that was still positive for SEOV in the RT-qPCR. Likely, this serum was taken too early after infection for neutralizing antibodies to develop.

**Table 4:** MNT_95_ titers of patient sera with a RT-PCR-confirmed orthohantavirus infection.

Patient ID	Country of Origin	RT-qPCR		Orthohantavirus MNT_95_					
				95% neutralization titer				Minimal Fold change	Conclusion
		+/−	Species	PUUV	TULV	DOBV	SEOV		
14-05	Netherlands	+	PUUV	1,280	<40	<40	<40	≥64	PUUV
14-08	Netherlands	+	PUUV	640	40	<40	<40	16	PUUV
16-07	Netherlands	+	PUUV	320	40	<40	<40	8	PUUV
17-02	Netherlands	+	PUUV	320	<40	<40	<40	≥16	PUUV
17-06b	Netherlands	+*	PUUV	1,280	<40	<40	<40	≥64	PUUV
17-13	Netherlands	+	PUUV	640	80	<40	<40	8	PUUV
17-14	Netherlands	+	PUUV	640	160	<40	<40	4	PUUV
17-23	Netherlands	+	PUUV	320	<40	<40	<40	≥16	PUUV
17-25b	Netherlands	+*	PUUV	≥5,120	640	<40	<40	≥8	PUUV
580	Slovenia	+	PUUV	320	<40	<40	<40	≥16	PUUV
17-07b	Netherlands	+*	SEOV	<40	<40	<40	<40	–	Negative
17-08	Netherlands	+	SEOV	<40	<40	<40	<40	–	Negative
306	Slovenia	+*	DOBV	<40	<40	80	<40	≥4	DOBV
465	Slovenia	+*	DOBV	<40	<40	40	<40	≥2	DOBV
625	Slovenia	+	DOBV	<40	<40	40	<40	≥2	DOBV
632	Slovenia	+*	DOBV	<40	<40	160	<40	≥8	DOBV
548	Slovenia	+	DOBV-Kurkino	<40	<40	160	<40	≥8	DOBV

Neutralization titers of ≥ 40 were considered positive and, when neutralizing titers are measured against multiple orthohantaviruses, a titer difference of ≥ 4-fold was required for definitive serotyping. The highest positive titer is highlighted in gray. For the samples marker with *, a paired sample to a previous RT-PCR-positive serum sample was tested in the microneutralization test (MNT).

### Validation: Sera From Human Patients With a Suspected DOBV Infection

In addition to the RT-PCR-confirmed rodent and human sera, a small serum panel (n=3, including two convalescent sera) from patients with a suspected DOBV infection was tested in the MNT. As expected, sera of all three patients were serotyped as DOBV infections (**Table 5**).

**Table 5:** MNT_95_ titers for different orthohantaviruses (DOBV)-suspected patient sera.

Serum #	Orthohantavirus MNT_95_					
	95% neutralization titer				Minimal Fold change	Conclusion
	PUUV	TULV	DOBV	SEOV		
DOBV suspected 1	< 40	< 40	320	< 40	≥ 16	DOBV
DOBV suspected 2	< 40	< 40	40	< 40	≥ 2	DOBV
DOBV suspected 3	40	40	640	80	8	DOBV

Neutralization titers of ≥ 40 were considered positive and, when neutralizing titers are measured against multiple orthohantaviruses, a titer difference of ≥ 4-fold was required for definitive serotyping. The highest positive titer is highlighted in gray.

### Validation: Dutch Blood Bank Cohort

As the incidence of orthohantavirus infections in Netherlands is relatively low (Sane et al., 2014), 85 randomly selected serum samples from healthy Dutch blood donors were included as negative controls during validation of the orthohantavirus MNT. As expected, all sera tested negative (data not shown), implying that neutralization is not caused by cytotoxic or virocidal components in serum, but is orthohantavirus specific.

### Evaluation: Diagnostic Cohort of Clinically Suspected Human Orthohantavirus Cases Diagnosed With an Orthohantavirus Infection Based on Routine Serology

To evaluate the MNT in a clinical diagnostic setting, a cohort consisting of 50 Dutch patients diagnosed with an orthohantavirus infection based on routine serology was tested in the comparative MNT. All sera (100%) showed neutralizing titers against at least one of the orthohantaviruses, and could thus be confirmed as orthohantavirus infections based on Gold Standard serology (**Table 6**). In addition, 47/50 sera (94%) could be serotyped conclusively using the MNT. The vast majority (44) were serotyped as PUUV infections, whereas three sera were found to be positive for SEOV. Without exclusion, the orthohantavirus that caused the infection as identified by MNT-based serotyping was part of the serogroup to which the highest titers were found during routine diagnostics using the mosaic IFA.

**Table 6:** MNT_95_ titers for the diagnostic cohort of clinically suspected human orthohantavirus cases diagnosed with an orthohantavirus infection based on routine serology.

Patient ID	Serum collection (days post disease onset)	Serogroup with highest reactivity in IFA	Orthohantavirus MNT_95_					
			95% neutralization titer				Minimal Fold change	Conclusion
			PUUV	TULV	DOBV	SEOV		
13-01b	> 32	DOBV/HTNV/SAAV/SEOV	<40	<40	<40	160	≥8	SEOV
13-02c	75	DOBV/HTNV/SAAV/SEOV	<40	<40	<40	160	≥8	SEOV
14-01	NA	PUUV/SNV	≥5120	40	<40	<40	≥128	PUUV
14-02	< 9	PUUV/SNV	1,280	320	<40	<40	4	PUUV
14-03	NA	PUUV/SNV	2,560	<40	<40	<40	≥128	PUUV
14-04b	> 53	PUUV/SNV	640	80	<40	<40	8	PUUV
14-06	NA	PUUV/SNV	2,560	40	<40	<40	64	PUUV
14-07	NA	PUUV/SNV	640	<40	<40	<40	≥32	PUUV
15-01	NA	PUUV/SNV	≥5,120	80	<40	<40	≥64	PUUV
15-02	7	ND	640	80	<40	<40	8	PUUV
15-03	10	PUUV/SNV	640	320	<40	<40	2	Inconclusive
15-04	6	PUUV/SNV	2,560	<40	<40	<40	≥128	PUUV
15-05	11	ND	640	80	<40	<40	8	PUUV
15-06	NA	PUUV/SNV	640	<40	<40	<40	≥32	PUUV
15-07	NA	PUUV/SNV	≥5,120	<40	<40	<40	>128	PUUV
16-01	< 3	PUUV/SNV	≥5,120	80	<40	<40	≥64	PUUV
16-02	7	ND	1,280	<40	<40	<40	≥64	PUUV
16-03	NA	PUUV/SNV	640	<40	<40	<40	≥32	PUUV
16-04	NA	PUUV/SNV	2,560	<40	<40	<40	≥128	PUUV
16-05	NA	PUUV/SNV	≥5,120	80	<40	<40	≥64	PUUV
16-06	NA	PUUV/SNV	1,280	<40	<40	<40	≥64	PUUV
16-08	NA	PUUV/SNV	160	<40	<40	<40	≥8	PUUV
16-09	NA	PUUV/SNV	320	<40	<40	<40	≥16	PUUV
16-10	12	PUUV/SNV	640	<40	<40	<40	≥32	PUUV
16-11	NA	PUUV/SNV	640	40	<40	<40	16	PUUV
16-12	NA	PUUV/SNV	160	80	<40	<40	2	Inconclusive
16-13	0	PUUV/SNV	≥5,120	40	<40	<40	≥128	PUUV
16-14	NA	PUUV/SNV	320	<40	<40	<40	≥16	PUUV
16-15b	30	DOBV/HTNV/SAAV/SEOV	<40	40	40	160	4	SEOV
16-16	NA	PUUV/SNV	2,560	40	<40	<40	64	PUUV
16-17	NA	PUUV/SNV	1,280	40	<40	<40	32	PUUV
16-18	NA	PUUV/SNV	320	<40	<40	<40	≥16	PUUV
17-01	11	ND	640	160	<40	<40	4	PUUV
17-03b	24	PUUV/SNV	640	40	<40	<40	8	PUUV
17-04b	NA	PUUV/SNV	640	<40	<40	<40	≥32	PUUV
17-05b	36	PUUV/SNV	640	<40	<40	<40	≥32	PUUV
17-09b	17	DOBV/HTNV/SAAV/SEOV	<40	80	<40	40	2	Inconclusive
17-10	NA	ND	160	<40	<40	<40	≥8	PUUV
17-11	NA	PUUV/SNV	640	<40	<40	<40	≥32	PUUV
17-12	NA	PUUV/SNV	640	<40	<40	<40	≥32	PUUV
17-15b	29	PUUV/SNV	1,280	40	<40	<40	32	PUUV
17-16	5	ND	2,560	<40	<40	<40	≥128	PUUV
17-17	NA	PUUV/SNV	2,560	40	<40	<40	64	PUUV
17-18	NA	ND	640	<40	<40	<40	≥32	PUUV
17-19b	43	PUUV/SNV	2,560	160	<40	<40	16	PUUV
17-20	1	PUUV/SNV	≥5,120	<40	<40	<40	>128	PUUV
17-21	NA	PUUV/SNV	1,280	<40	<40	<40	≥64	PUUV
17-22	8	ND	2,560	<40	<40	<40	≥128	PUUV
17-24	NA	PUUV/SNV	640	40	<40	<40	16	PUUV
17-26	NA	ND	1,280	<40	<40	<40	≥64	PUUV

Neutralization titers of ≥ 40 were considered positive and, when neutralizing titers are measured against multiple orthohantaviruses, a titer difference of ≥ 4-fold was required for definitive serotyping. The highest positive titer is highlighted in gray.

Of the sera that could not be serotyped, two sera (15-03 and 16-12) showed a neutralizing titer for both PUUV and TULV with only a two-fold difference, and were thus considered inconclusive. A third inconclusive serum (17-09b) showed a rather atypical neutralization profile of cross-neutralization between the different serogroups (both TULV and SEOV were positive with MNT_95_ titers of 80 and 40, respectively) while no cross-neutralization within the serogroups was observed (both DOBV and PUUV were negative). Most likely this patient was infected with SEOV, as the patient owned SEOV-positive rats.

## Discussion

Here, we describe the successful development and implementation of a MNT including all four European human pathogenic orthohantavirus species. In a clinical cohort of orthohantavirus patients, all patients could be confirmed, with conclusive identification of the causative orthohantavirus species for 94% of the patients. Our data shows that the orthohantavirus MNT represents a more high-throughput alternative to the orthohantavirus PRNT (the Gold Standard in orthohantavirus VNTs) for serotyping orthohantavirus infections. This is in line with two previous studies (Horling et al., 1992; Li et al., 2017) that showed that the MNT, albeit in slightly different set-ups, was as a viable alternative to the PRNT for the detection of PUUV neutralizing antibodies (Horling et al., 1992) and serotyping human HTNV and SEOV infections (Li et al., 2017).

The MNT was validated using 17 rodent (**Table 3**) and 17 human sera of RT-PCR-confirmed infections (**Table 4**), with the addition of three human sera of suspected DOBV infections (**Table 5**). For the rodent sera, 16/17 sera were serotyped in line with RT-PCR outcomes, while one DOBV-Kurkino positive *A. agrarius* serum remained negative in the MNT (**Table 3** - rDOBV4). As this serum only showed low IgG levels in the IFT, it is possible that the serum was taken too early after infection for neutralizing antibodies to form.

All PUUV- and DOBV-confirmed/suspected human sera were serotyped as expected. However, both RT-qPCR-confirmed SEOV sera remained negative in the MNT at 95% cut-off (**Table 4** - 17-07b and 17-08). One of the serum samples tested was from a patient that was still viremic (17-08). For the second patient, a paired serum to the RT-qPCR positive serum was tested, which was expected to be early convalescent but for which the exact period between onset of disease and serum sampling was not known (17-07b). This serum sample did show some neutralizing potential at the 1:40 and 1:80 dilutions, but never reached the cut-off of 95% neutralization. However, the assay proved its worth for confirmation of SEOV infections as three sera in the clinical evaluation cohort could be serotyped as SEOV infections (**Table 6** - 13-01b, 13-02c, and 16-15b). Interestingly, all three positive sera were convalescent, taken > 1 month after onset of disease. Additionally, all three sera showed relatively high IgG titers against the *Murinae*-borne orthohantaviruses in the diagnostic IFA (2- to 16-fold higher than serum 17-07b). For both PUUV and DOBV neutralizing antibodies could already be detected in the first few days after infection (**Table 4** and (Horling et al., 1992)). These data, albeit limited, might suggest that neutralizing antibodies become detectable only relatively late after SEOV infection, implicating the need to test convalescent sera to confirm SEOV infections by MNT. However, solid evidence for these differences in kinetics can only be provided by extensive studies.

Lundkvist et al. described that acute and early convalescent human serum samples (taken < 1 month post onset of disease) might be difficult to serotype due to high cross-reactivity in the VNT (Lundkvist et al., 1997). Here, relatively few problems with cross-reactivity were encountered in both the validation and evaluation. Using 11 acute and 4 possibly early convalescent human sera, cross-reactivity was only seen to a low extent and, if present, primarily within the serogroups. Only for one serum sample (**Table 5** - DOBV suspected 3), cross-neutralization toward the other serogroup was observed. This sample showed a relatively high neutralizing titer toward the infecting virus (MNT_95_ of 640, the highest neutralizing titer measured against DOBV in this study), which might explain the low levels of cross-neutralization toward PUUV and TULV. This explanation is supported by the notion that also *R. norvegicus* sera with high neutralizing titers against SEOV (≥1280) showed some degree of cross-neutralization toward PUUV. Interestingly, however, cross-neutralization from the *Arvicolinae*-borne orthohantaviruses (PUUV and TULV) toward the *Murinae*-borne orthohantaviruses (DOBV and SEOV) was not observed during validation, even though some rodent and human sera had neutralizing titers of ≥5120 toward either PUUV or TULV. It was, however, observed once in the diagnostic cohort (**Table 6** - 17-09b) for which we have no clear explanation, other than the relatively early sampling date of the serum tested (17 days post onset of disease), which might have affected serotyping (Lundkvist et al., 1997).

The vast majority of cases (44/50) in our clinical cohort of orthohantavirus infections were identified as caused by PUUV, which confirms the assumption that PUUV is the major cause of orthohantavirus infections in Netherlands (Goeijenbier et al., 2014; Sane et al., 2014). Three human SEOV infections were confirmed of which one (**Table 6** - 16-15) was previously described as the first proven SEOV case in Netherlands based on IFT serology and an epidemiological link to SEOV RNA positive rats (Swanink et al., 2018), and now confirmed by comparative VNT. Interestingly, the two other SEOV cases (**Table 6**- 13-01 and 13-02) confirmed in this study predated this case by almost three years, but were not recognized as SEOV cases using routine serological diagnostic assays (ELISA and IFA) at the time. This underlines the added value of the use of comparative MNT for determination of causative orthohantavirus species. It must however be noted that evidence of human SEOV infections in Netherlands was already observed in the 1980s (Groen et al., 1991; Clement et al., 2019), however these cases were never confirmed by either RT-PCR or VNT.

No evidence for DOBV and TULV infections was found in the 50 patients included in our diagnostic cohort. DOBV infections are only known as imported cases (Geurts van Kessel et al., 2016). Its main reservoir (*A. flavicollis*) has only limited presence in Netherlands and is considered not to carry the virus. TULV, on the other hand, is circulating among the common vole populations at multiple locations in Netherlands (Reusken et al., 2008; Maas et al., 2017). However, no human TULV infections have been recognized in Netherlands so far, which is in line with the only sporadic human TULV cases reported in Europe to date (Schultze et al., 2002; Klempa et al., 2003; Zelena et al., 2013; Reynes et al., 2015), even though the virus is able to infect humans (Vapalahti et al., 1996; Mertens et al., 2011). To gain insight in the infection pressure of TULV in Netherlands dedicated seroprevalence studies are needed for which our developed MNT represents a crucial tool. Unfortunately, no human sera of the few TULV cases in Europe could be tested in the MNT. As sera from TULV-positive rodents could be clearly discerned from the other orthohantavirus-positive rodent sera using the comparative MNT, we expect that the assay is able to correctly serotype TULV-positive human sera as well. However, the exact performance of the MNT with human TULV sera remains to be tested.

In conclusion, we demonstrate the successful development and clinical implementation of a comparative European orthohantavirus MNT to determine the infecting virus species in European HFRS/NE patients. Identification of the causative species is needed for an adequate Public Health response, e.g. in the case of SEOV and pet rats, and can support individual patient care. The general absence of orthohantavirus neutralization tests in European reference laboratories (EVD-LabNet) illustrates that the implementation of virus neutralization tests for orthohantaviruses is not a straight forward procedure. We will address this issue by the roll-out of this test, including across laboratory performance comparisons, to multiple European laboratories (EVD-LabNet, pers. comm.) to support patient diagnostics, surveillance and Public Health responses.

## Data Availability Statement

The raw data supporting the conclusions of this article will be made available by the authors upon request, without undue reservation.

## Ethics Statement

Ethical review and approval was not required for the study on human participants in accordance with the local legislation and institutional requirements. The patients/participants provided their written informed consent to participate in this study. The animal study was reviewed and approved by Dutch (DEC project numbers 200900164 and 201200208) and Slovenian Animal Ethics Committee (Administration for Food Safety, Veterinary Sector and Plant Protection: U34401-15/2018/8).

## Author Contributions

TH, JR, and CR contributed to the design of the study. TH, IZ, MH, and AV performed the experiments. JR, MM, TA-Ž, AV, and MK provided critical samples for validation and assessment of the assay. TH and CR wrote the manuscript. All authors contributed to the article and approved the submitted version.

## Conflict of Interest

The authors declare that the research was conducted in the absence of any commercial or financial relationships that could be construed as a potential conflict of interest.
