# Supplementary material for: Development of a Comparative European Orthohantavirus Microneutralization Assay With Multi- Species Validation and Evaluation in a Human Diagnostic Cohort
Source: Front Cell Infect Microbiol. 2020 Dec 22;10:580478. doi: 10.3389/fcimb.2020.580478 (PMC7783042; doi:10.3389/fcimb.2020.580478)
Supplement: Supplementary file 1 [file DataSheet_1.pdf]

Supplementary Material

Development of a comparative European orthohantavirus microneutralization assay with multi-species validation and evaluation in a human diagnostic cohort.

Tabitha E. Hoornweg, Ilse Zutt, Ankje de Vries, Miriam Maas, Marieke N. Hoogerwerf, Tatjana Avšič-Županc, Miša Korva, Johan H.J. Reimerink, Chantal B.E.M. Reusken

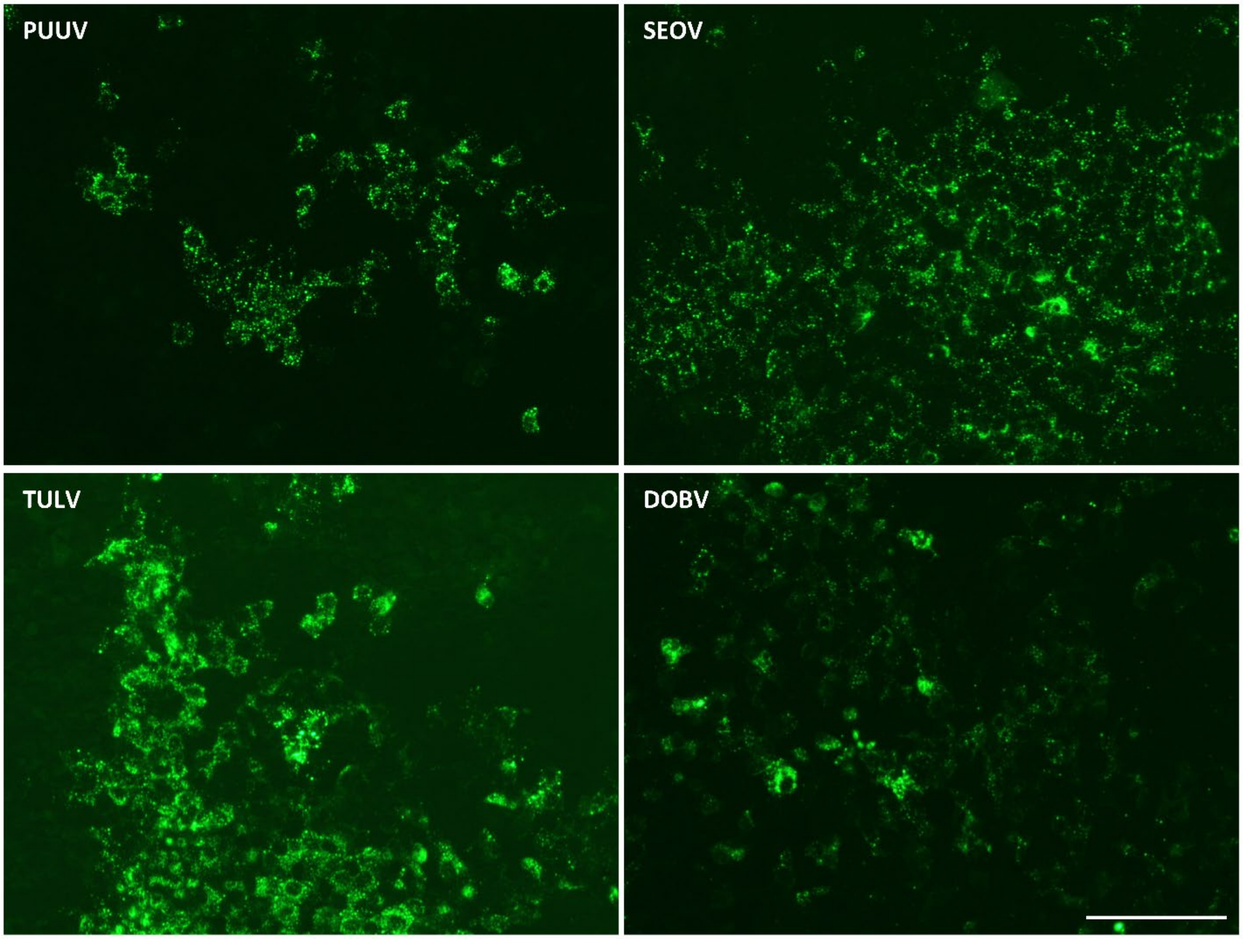

Supplementary Figure 1. Immunofluorescent staining of Orthohantavirus-infected Vero-E6 cells. All images were adjusted is a similar manner for visual purposes. Scale bar indicates 100 µm.
